# “Adjuvant Radioactive iodine 133 ablation in papillary microcarcinoma of thyroid: Saudi Arabian experience”

**DOI:** 10.1186/s40463-015-0108-0

**Published:** 2015-12-01

**Authors:** Khalid Hussain AL-Qahtani, Mushabbab Al Asiri, Mutahir A. Tunio, Naji J. Aljohani, Yasser Bayoumi, Hanadi Fatani, Abdulrehman AlHadab

**Affiliations:** Department of Otolaryngology-Head & Neck Surgery, College of Medicine, King Saud University, Riyadh, Saudi Arabia; Radiation Oncology, Comprehensive Cancer Center, King Fahad Medical City, Riyadh, 59046 Saudi Arabia; Endocrinology and thyroid Oncology, King Fahad Medical City, Riyadh, 59046 Saudi Arabia; Radiation Oncology, NCI, Cairo University, Cairo, Egypt; Histopathology, King Fahad Medical City, Riyadh, 59046 Saudi Arabia; Radiation Oncology, King AbdulAziz University, Riyadh, 59046 Saudi Arabia

**Keywords:** Papillary microcarcinoma, Optimal treatment, Adjuvant radioiodine ablation, Disease free survival, Saudi Population

## Abstract

**Background:**

Papillary Microcarcinoma (PMC) of thyroid is a rare type of differentiated thyroid cancer (DTC), which according to the World Health Organization measures 1.0 cm or less. The gold standard of treatment of PMC is still controversy. Our aim was to contribute in resolving the debate on the therapeutic choices of the surgical and adjuvant I-131 (RAI) treatment in PMC.

**Methods:**

From 2000 to 2012, 326 patients were found to have PMC and were retrospectively reviewed for clinicopathological characteristics, treatment outcomes and prognostic factors.

**Results:**

Mean age of cohort was 42.6 years (range: 18–76) and the mean tumor size was 0.61 cm ± 0.24; lymph node involvement was seen in 12.9 % of cases. Median follow up period was 8.05 years (1.62–11.4). Total 23 all site recurrences (7.13 %) were observed; more observed in patients without I-131 ablation (*p <0.0001*). Ten year DFS rates were 89.6 %. Cox regression Model analysis revealed size, histopathologic variants, multifocality, extrathyroidal extension, lymphovascular space invasion, nodal status, and adjuvant RAI ablation the important prognostic factors affecting DFS.

**Discussion:**

Despite excellent DFS rates, a small proportion of patients with PMC develop recurrences after treatment. Adjuvant RAI therapy improves DFS in PMC patients with aggressive histopathologic variants, multifocality, ETE, LVSI, tumor size (> 0.5 cm) and lymph node involvement. Failure of RAI ablation to decrease risk in N1a/b supports prophylactic central neck dissection during thyroidectomy, however more trials are warranted.

**Conclusion:**

Adjuvant I-131 ablation following thyroidectomy in PMC patients, particularly with poor prognostic factors improves DFS rates.

## Background

In Saudi Arabia, the incidence of differentiated thyroid cancers (DTC) especially papillary thyroid cancers (PTC) is increasing exponentially over the past years accounting for more than 10 % of all cancers among females [1, 2]. Higher rates for identification of PTC in recent years are attributed to the use of high resolution neck ultrasonography (USG) and USG-guided fine needle aspiration biopsy (FNAB) [3]. With the use of these high resolution transducers, papillary microcarcinoma (PMC), i.e. tumor size 1 cm or less can easily be detected [4, 5]. Patients with PMC have generally an excellent outlook with use of surgery, radioactive iodine-131 (RAI) ablation, suppression of thyroid-stimulating hormone (TSH) secretion with levothyroxine, with long term disease-free survival (DFS) of 84–97 % [6]. However, still there is much debate regarding the most appropriate treatment of PMC ranging from observation alone to over-treatment with surgery followed by adjuvant RAI ablation [7–10].

In the present study, we aimed to evaluate the different prognostic factors for DFS in PMC patients in our population, and also to determine the DFS in patients with PMC treated with or without adjuvant RAI ablation following thyroidectomy.

## Methods

After formal approval from the institutional ethical committee, medical records of 1192 patients with confirmed papillary thyroid cancers (PTC) who were treated or followed up in two major referral hospitals of Riyadh, Saudi Arabia, during the period of July 2000 and December 2012 were reviewed using computer based departmental database system. Patients with PMC were retrieved in following manner;

### Definition

PMC was defined according WHO classification system for thyroid tumors as “PTC is measuring ≤ 1 cm in greatest dimension” [5].

### Demographic, clinicopathological and radiological variables

Demographic and clinical data including age at the diagnosis, gender, and symptomatology were reviewed. A detailed second review of all histopathological specimens was performed by experienced histopathologist. Different histopathological parameters, including the location of tumor, tumor size, histopathologic variants, multifocality, extrathyroidal extension (ETE), lymphovascular space invasion (LVSI), surgical margin status, and cervical lymph node status and background thyroid tissue were also recorded. Data from different imaging modalities, including neck ultrasonography, whole body I-131 scintigraphy (WBS), computed tomography (CT) scan of neck and chest, flourodeoxyglucose positron emission tomography (FDG-PET) was collected. Periodic postoperative thyroid function tests (TFTs), thyroid antibodies and stimulated thyroglobulin (TG) levels (off thyroxin or thyrotropin-Alfa injection) were also reviewed. Different treatment modalities, including hemi-thyroidectomy (removal of lobe and isthmus), total thyroidectomy (removal of entire gland), neck dissection, adjuvant RAI ablation, different doses used in millicurie (mCi) and the details of neck irradiation details (if given) were also reviewed.

The primary endpoint was the disease free survival (DFS). Secondary points were; the frequency of PMC and histologic variants, local recurrence free survival (LRFS), distant metastasis free survival (DMFS) and overall survival (OS) according to (a) treatment with or without adjuvant I-131 ablation and (b) according to primary tumor size (≤0.5 cm vs. > 0.5 cm).

Local recurrence was defined as, clinically or radiologically detectable recurrences in the thyroid bed or in cervical lymph nodes on imaging (ultrasonography, WBS and CT-PET) after evaluating for elevated thyroglobulin (TG) levels. Distant metastasis was defined as, clinically or radiologically detectable disease outside the neck on imaging (WBS, CT imaging and CT-PET) after evaluating for elevated thyroglobulin (TG) levels. The DFS was defined as, the duration between the surgery date and the date of documented disease reappearance/relapse, death from cancer and/or last follow-up (censored). The OS was defined as, the duration between the surgery date and the date of patient death or last follow-up (censored).

### Statistical analysis

Chi-square test, Student’s *t* test, or Fisher exact tests were used to determine the differences in various clinical variables. Multivariate logistic regression was done using Cox proportional hazards modeling. Probabilities of LRFS, DMFS, DFS and OS were shown with the Kaplan-Meier method and the comparisons for various survival curves were performed using log rank. All statistical analyses were performed using the computer program SPSS version 16.0.

## Results

### Demographic and clinicopathological features of cohort

Among the 1192 PTC patients in our departmental database, 377 (31.6 %) patients were found to have PMC. Fifty one (13.3 %) patients with insufficient data regarding size, treatment and follow-up period were excluded. The remaining study cohort (*n* = 326) consisted of 271 (83.1 %) women and 55 (16.9 %) men; the median age at diagnosis was 42.6 years ±11.6. The majority of patients had total thyroidectomy (*n* = 299, 91.7 %); only 27 (8.3 %) patients underwent lobectomy. The mean tumor size was 0.61 cm ± 0.24, with 12.9 % (*n* = 42) involvement of cervical lymph nodes (level VI in 34 patients). The predominant histopathologic variants were, classic (265 patients), follicular (41 patients), and tall cell (11 patients). Other clinicopathological features are described in Table 1.

**Table 1:** Patients characteristics

Variable	Whole cohort *N* (%)	RAI ablation *N* (%)	Without RAI ablation *N* (%)	*P* value*
Total patients	326/1192 (27.4 %)	182/326 (55.8)	144/326 (44.2)	*0.06*
Age (years)	42.6 (18–76) SD ±11.6	43.2 (18–76) SD ± 12.4	41.8 (19–71) SD ± 10.2	
≤45 years	201 (61.7)	110 (60.4)	94 (65.3)	*0.81*
≥45 years	125 (38.3)	72 (39.6)	50 (34.7)	
Gender				
Female	271 (83.1)	146 (80.2)	125 (86.8)	*0.06*
Male	55 (16.9)	36 (19.8)	19 (13.2)	
Female to male ratio	4.9	4.0	6.5	
Type of surgery				
Total thyroidectomy	299 (91.7)	182 (100)	117 (81.3)	*0.04*
Hemi-thyroidectomy	27 (8.3)	-	27 (18.7)	
Lymph node surgery				
Central neck dissection	88 (27.0)	54 (29.7)	34 (23.6)	
Lateral neck dissection	18 (5.5)	9 (4.9)	9 (6.3)	0.9
Sampling	55 (16.9)	25 (13.7)	30 (20.8)	
None	165 (50.6)	94 (51.7)	71 (49.3)	
Mean size (cm)	0.61 (0.1–1.0) ± 0.24	0.72 (0.2–1.0) ± 0.21	0.44 (0.1–0.9) ± 0.2	
≤0.5 cm	161 (49.4)	50 (27.5)	111 (77.1)	*<0.0001*
≥0.5 cm	165 (50.6)	132 (72.5)	33 (22.9)	
Histopathologic variants				
Classic	265 (81.3)	143 (78.6)	122 (84.7)	
Follicular	41 (12.6)	21 (11.5)	20 (13.9)	
Hurthle cell	8 (2.5)	6 (3.3)	2 (1.4)	
Tall cell	11 (3.4)	11 (6.0)	-	*0.001*
Sclerosing	1 (0.3)	1 (0.5)	-	
Multifocal				
Yes	125 (38.3)	122 (67.1)	3 (2.1)	*<0.0001*
No	201 (61.7)	60 (32.9)	141 (97.9)	
ETE				
Yes	62 (19.0)	57 (31.3)	5 (3.5)	*<0.0001*
No	264 (81.0)	125 (68.7)	139 (96.5)	
LVSI				
Yes	55 (16.9)	49 (26.9)	6 (4.2)	*<0.0001*
No	271 (83.1)	133 (73.1)	138 (95.8)	
Surgical margins				
Positive	35 (10.7)	30 (16.5)	5 (3.5)	*<0.0001*
Negative	291 (89.3)	152 (83.5)	139 (96.5)	
Lymph node metastasis				
Yes	42 (12.9)	42 (23.1)	-	*<0.0001*
N1a	34 (73.8)	34 (73.8)		
N1b	8 (19.2)	8 (19.2)		
No	284 (87.1)	140 (76.9)	144 (100)	
Background thyroid tissue				
Normal	98 (30.1)	47 (25.8)	51 (35.4)	
Multi-nodular goiter	106 (32.5)	60 (32.9)	46 (31.9)	
Lymphocytic thyroiditis/Hashimotos’ thyroiditis	122 (37.5)	75 (41.3)	47 (32.6)	*0.052*
Distant Metastasis at presentation	3 (0.9)	3 (1.65)	-	*<0.0001*
AJCC staging				
I	217 (66.5)	73 (40.1)	139 (96.5)	
II	-	-	-	
III	96 (29.5)	96 (52.6)	5 (3.5)	*<0.0001*
IVA	10 (3.1)	10 (5.6)	-	
IVB	-	-	-	
IVC	3 (0.9)	3 (1.7)	-	
Mean postoperative TG (ng/ml)	1.39 (0.1–42890)	2.44 (0.1–42890)	0.39 (0.1–8.9)	*0.62*
RAI dose				
30 mCi		50 (27.5)	-	<0.0001
100 mCi		85 (46.7)	-	
150-200 mCi		47 (25.8)	-	
RT to Neck	2 (0.61)	2 (1.1)	-	*<0.0001*
Recurrences				
Locoregional	13 (3.9)	4 (2.2)	9 (6.2)	*<0.001*
Distant	10 (3.1)	4 (2.2)	6 (4.2)	

*P value pertaining to the variation in clinicopathological characteristics between two groups

*RAI* radioactive iodine 131, *N* number, *SD* standard deviation, *ETE* extra-thyroidal extension, *LVSI* lymphovascular space invasion, *AJCC* American joint committee on cancer, *TG* thyroglobulin, *mCi* millicurie, *RT* radiation therapy

### Clinicopathological features and DFS Comparison in PMC patients treated with and without I-131 ablation

Among 326 patients, 182 (55.8 %) patients were given adjuvant RAI ablation as shown in Table 1. Major indications for adjuvant RAI ablation were multifocality (67.1 %), extra-thyroidal extension (ETE) in 31.3 % of cases, aggressive histopathologic variants (tall cell, sclerosing), lymph node metastasis (23.1 %) and distant metastasis at time of presentation (1.65 %). Primary tumor size was not a primary indication in our series; however the observed mean tumor size was bigger in patients treated with adjuvant RAI ablation (0.72 cm vs. 0.44 cm). RAI ablation doses were as; 30 m-curie (mCi) for tumors with multifocality and focal ETE (27.5 %); 100 mCi for tumors with multifocality, ETE, LVSI, positive surgical margins, poor histopathologic variants, and elevated postoperative stimulated TG levels (>2 ng/ml) (46.7 %); 150 mCi for positive lymph nodes (24.7 %), and 200 mCi for distant metastasis at the time of diagnosis (1.65 %). RAI ablation was tolerated well without any grade 3 or 4 side effects. Additional neck irradiation was given in two patients with adherent tumors; trachea (one patient; 0.5 %) and skeletal muscle (one patient; 0.5 %).

A median follow-up period was 8.05 years (range: 1.62–11.4). For whole cohort, the 5 and 10 years LRFS were 98.4 % and 96.8 % respectively; DMFS rates were 92.4 % at 5 years and 90 % at 10 years. Five and 10 years OS rates were 99.3 % and 98.6 % (two deaths) and the 5 and 10 years DFS rates were 94.7 % and 89.6 %.

**Fig. 1:**
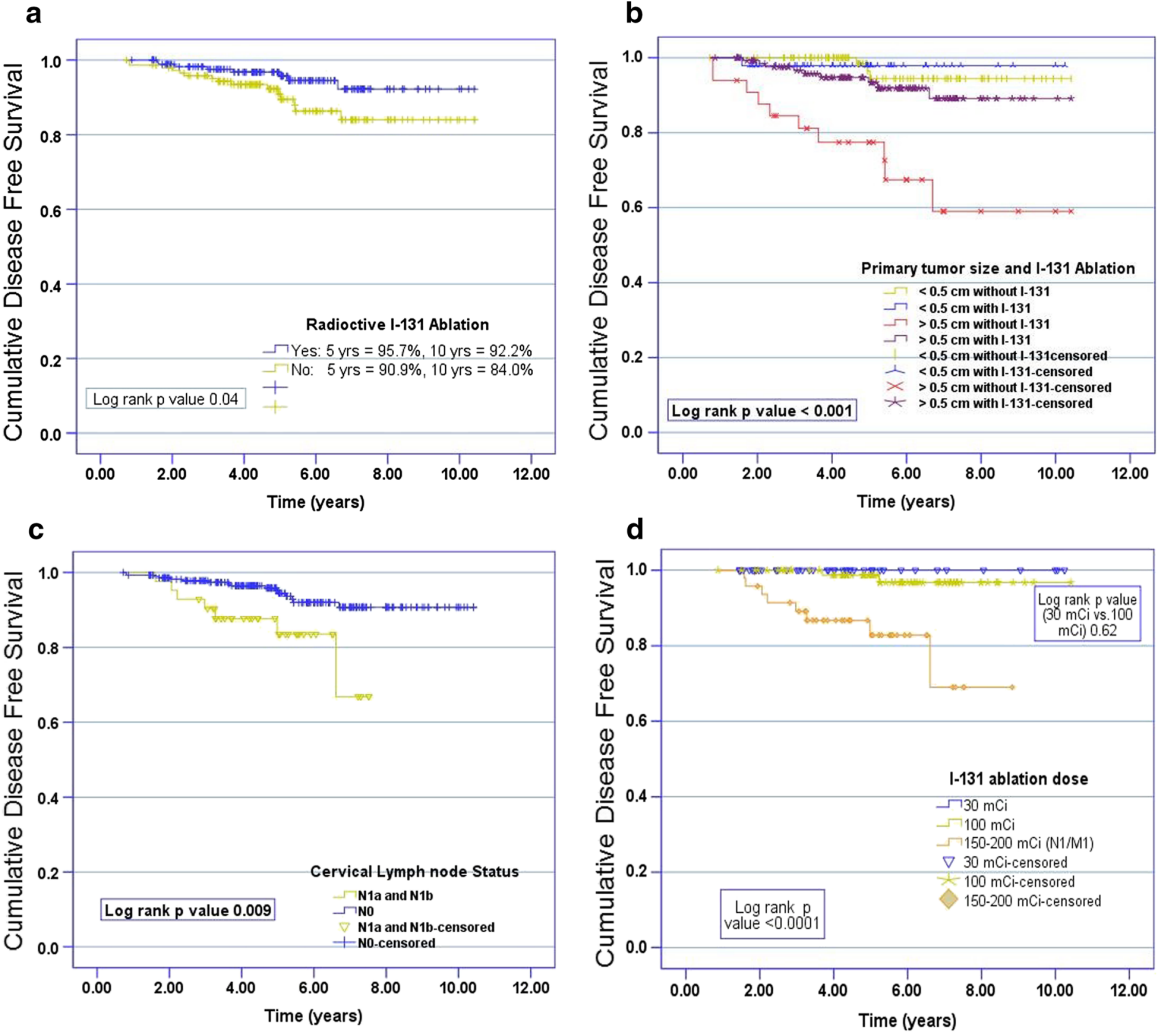
Kaplan-Meier curves of disease free survival (**a**) according to treated with or without radioactive iodine 131 ablation, (**b**) primary size (≤0.5 cm vs. > 0.5 cm) treated with or without radioactive iodine 131 ablation, (**c**) lymph node status and (**d**) dose regimens (30 mCi vs. 100 mCi)

Total 23 recurrences (7.13 %) were observed; 8/182 in patients with RAI ablation and 15/144 in patients without RAI ablation. The pattern of recurrences was as: three patients had disease in thyroid bed only, 10 had cervical nodes, and 10 failed at distant sites (9 patients in lungs and one patient in bones). Combined locoregional and distant recurrences were seen in 3 patients. The elevated TG levels were always seen with local recurrences and distant metastasis. The isolated locoregional recurrences were salvaged by surgery (lateral neck dissection; 7 patients, completion thyroidectomy; 2 patients and excision in one patient), followed by RAI ablation (12 patients) and distant failures were salvaged by RAI ablation (9 patients) and palliative irradiation for bony lesion (one patient). Time to initial local recurrence was 0.8 years and time to initial distant metastasis was 1.5 year. The 5 and 10 year DFS rates were 95.7 % vs. 90.9 % and 92.2 % vs. 84 % in patients with and without RAI ablation respectively (*p* = *0.04*) Fig. 1a. The 5 and 10 year DFS rates according to different prognostic factors are summarized in Table 2. The overall 5 and 10 year DFS rates were significantly dropped in the presence of poor histopathologic variants (*p < 0.001*) and ETE. In addition to these factors, multifocality (*p* < 0.001) LVSI (*p* = 0.001) and elevated postoperative thyroglobulin levels > 2 ng/ml (*p* = 0.04) resulted in inferior 5 and 10 year DFS in patients treated without RAI ablation.

**Table 2:** Disease free survival according to different prognostic factors

Variable	RAI ablation				Without RAI ablation			
	5 years-DFS	*p*	10 years-DFS	*p*	5 years-DFS	*p*	10 years-DFS	*p*
Age								
≤45 years	97.8 %		93.5 %		87.6 %		83.6 %	
≥45 years	92.5 %	*NS*	85.3 %	*NS*	88.5 %	*NS*	84.5 %	*NS*
Gender								
Female	95.4 %		91.2 %		93.8 %		82.2 %	
Male	96.8 %	*NS*	89.4 %	*NS*	90.4 %	*NS*	82.0 %	*NS*
Histopathologic variants								
Classic	96.1 %		93.5 %		95.5 %		92.3 %	
Follicular	94.7 %		90.9 %		78.9 %		59.6 %	
Hurthle cell	96.1 %		92.7 %		90.0 %	*0.001*	78.9 %	*<0.001*
Tall cell	68.2 %		-			-		
Sclerosing	55.0 %	*0.002*	-	*<0.001*		-		
Multifocal								
Yes	95.2 %		90.9 %		66.7 %		33.3 %	
No	96.6 %	*NS*	93.4 %	*NS*	90.0 %	*<0.001*	88.3 %	*<0.0001*
Surgical margins								
Positive	96.6 %		91.5 %		86.3 %		84.0 %	
Negative	96.8 %	*NS*	95.3 %	*NS*	93.2 %	*NS*	87.9 %	*NS*
ETE								
Yes	89.5 %		85.5 %		40.0 %		0.0 %	
No	98.2 %	*0.03*	95.1 %	*0.02*	91.7 %	*<0.0001*	80.1 %	*<0.0001*
LVSI								
Yes	89.5 %		85.5 %		80.0 %		60.0 %	
No	92.5 %	*NS*	89.4 %	*NS*	93.2 %	*0.02*	84.4 %	*0.001*
Mean postoperative TG								
≤2 ng/ml	96.6 %		93.4 %		93.2 %		91.7 %	
>2 ng/ml	89.5 %	*NS*	85.5 %	*NS*	87.9 %	*NS*	80.1 %	*0.04*
Surgery								
Total thyroidectomy	96.0 %		94.4 %		93.2 %		87.5 %	
Hemi-thyroidectomy	91.5 %	*0.03*	86.0 %	*0.03*	85.1 %	*0.04*	80.2 %	*0.02*

*RAI* radioactive iodine 131, *yr* year, *DFS* disease free survival, *SD* standard deviation, *ETE* extra-thyroidal extension, *LVSI* lymphovascular space invasion, *TG* thyroglobulin

### Clinicopathological features and DFS comparison among PMC of size ≤ 0.5 cm and > 0.5 cm

With regard to the difference in DFS (locoregional and distant failure), a comparative analysis was performed according to primary tumor size (≤0.5 cm vs. > 0.5 cm) as described in the Table 3. About 161 (49.4 %) patients had tumors of size ≤ 0.5 cm and 165 (50.6 %) patients had tumors of size above 0.5 cm in greatest dimension. Significant demographic and clinicopathological differences were observed between two groups. Patients with tumor size ≤ 0.5 cm were younger (mean age 36.7 years), with higher female to male ratio (6.3), and with more aggressive histopathologic variants (tall cell, sclerosing). The cervical lymph node metastases were seen in 9.3 % of patients with tumor size ≤ 0.5 cm as compared to patients with tumor size > 0.5 cm (16.4 %) with p < 0.001. Patients with tumor size ≤ 0.5 cm had high rates of hemi-thyroidectomy (18.7 %), less adjuvant RAI ablation (31.1 %) with low recurrence rates. There was also no significant difference in 5 and 10 year DFS rates in in patients with tumor size ≤ 0.5 cm treated with or without adjuvant RAI ablation (*p = 0.71*) Fig. 1b. Further it was seen that adjuvant RAI ablation did better in N0 as compared to N1 neck status Fig. 1c. Also in patients treated with adjuvant RAI ablation, no significant difference was observed between two dose regimens (30 mCi vs. 100 mCi) with *p* = 0.62 (Fig. 1d).

**Table 3:** Comparative analysis of clinicopathological characteristics based on the size of primary tumors

Variable	Tumor size ≤ 0.5 cm *N* (%)	Tumor size > 0.5 cm *N* (%)	*P* value
Total patients	161/326 (49.4)	165/326 (50.6)	-
Age (years)	36.7 (8–71)	47.8 (8–76)	
≤45 years	107 (66.5)	94 (56.9)	*0.034*
≥45 years	54 (33.5)	71 (43.1)	
Gender			
Female	139 (86.4)	132 (80.0)	*0.08*
Male	22 (13.6)	33 (20.0)	
Mean size (cm)	0.38 (0.1–0.5)	0.68 (0.6–1.0)	*<0.001*
Histopathologic variants			
Classic	126 (78.2)	139 (84.3)	
Follicular	26 (16.2)	14 (8.5)	
Hurthle cell	4 (2.5)	4 (2.4)	0.023
Tall cell	4 (2.5)	7 (4.3)	
Sclerosing	1 (0.6)	-	
Multifocal			
Yes	36 (22.4)	89 (53.9)	
No	125 (77.6)	76 (46.1)	*<0.001*
ETE			
Yes	16 (9.9)	46 (27.9)	*<0.001*
No	145 (90.1)	119 (72.1)	
LVSI			
Yes	14 (8.7)	41 (24.9)	*<0.001*
No	147 (91.3)	124 (75.1)	
Surgical margins			
Positive	5 (3.1)	30 (18.2)	
Negative	156 (96.9)	135 (81.8)	*<0.001*
Background thyroid tissue			
Normal	60 (37.3)	38 (23.0)	
Multi-nodular goiter	48 (29.8)	58 (35.2)	
Lymphocytic thyroiditis/Hashimotos’ thyroiditis	53 (32.9)	69 (41.8)	*0.05*
Lymph node metastasis			
Yes	15 (9.3)	27 (16.4)	
No	146 (90.7)	138 (83.6)	*<0.001*
RAI ablation			
Yes	50 (31.1)	132 (67.3)	*<0.001*
No	111 (68.9)	22 (13.3)	
Recurrences			
Locoregional	4 (2.5)	9 (5.5)	
* Thyroid bed*	*1/4*	*2/9*	
* Lymph nodes*	*3/4*	*7/9*	*<0.001*
Distant	2 (1.3)	8 (4.9)	
* Lungs*	*2*	*7/8*	
* Bone*	-	*1/8*	

*I-131* radioactive iodine 131, *N* number, *ETE* extra-thyroidal extension, *LVSI* lymphovascular space invasion, *RAI* radioactive iodine

### Prognostic factors

Cox regression Model using univariate and multivariate analysis for DFS to predict important prognostic factors Table 4. Important prognostic factors were, histopathologic variants (*p < 0.0001*), multifocality (*p* < 0.0001), ETE (*p < 0.0001*), LVSI (*p =0.03*), nodal status (*p* < 0.0001), and adjuvant RAI ablation (*p* < 0.0001).

**Table 4:** Cox regression model of various prognostic factors for disease specific survival

Variable	All patients			
	Univariate analysis		Multivariate analysis	
	RR (95 % CI)	*p*	RR (95 % CI)	*p*
Age				
≤45 years	1.05 (0.7–1.3)		1.07 (0.8–1.3)	
≥45 years	1.10 (0.8–1.4)	*0.6*	1.10 (0.9–1.3)	*0.06*
Gender				
Female	1.07 (0.9–1.4)		1.05 (0.7–1.3)	
Male	1.05 (0.7–1.3)	*0.6*	1.40 (1.2–1.6)	*0.05*
Histopathologic variants				
Classic	1.05 (0.7–1.2)		1.20 (0.8–1.6)	
Follicular	1.00 (0.6–1.8)		1.18 (0.7–1.5)	
Hurthle cell	1.30 (1.1–1.7)		2.00 (1.6–2.4)	
Tall cell	2.70 (1.6–4.5)		2.82 (2.4–4.6)	
Sclerosing	1.80 (1.6–2.9)	<*0.0001*	2.00 (1.6–3.0)	<*0.0001*
Multifocal				
Yes	3.1 (2.8–4.2)		2.94 (2.2–3.4)	
No	1.0 (0.8–1.2)	*<0.0001*	1.07 (0.9–1.3)	*<0.0001*
Surgical margins				
Positive	1.10 (0.9–1.4)		1.20 (0.8–1.6)	
Negative	1.07 (0.9–1.4)	*0.7*	1.17 (0.6–1.2)	*0.68*
ETE				
Yes	4.2 (3.5–5.1)		3.31 (1.7–4.2)	
No	1.05 (0.7–1.1)	*<0.0001*	1.17 (0.9–1.4)	*<0.0001*
LVSI				
Yes	2.0 (1.7–2.9)		1.81 (1.6–2.8)	
No	1.0 (0.8–1.2)	*0.02*	1.04 (0.9–1.5)	*0.03*
Lymph nodes				
Positive	4.45 (3.7–6.8)		3.74 (3.4–5.9)	
Negative	1.17 (0.9–1.4)	*<0.0001*	1.01 (0.8–1.3)	*<0.0001*
Mean postoperative TG				
≤2 ng/ml	1.01 (0.7–1.2)		1.05 (0.7–1.2)	
>2 ng/ml	1.04 (0.9–1.5)	*0.6*	1.00 (0.6–1.8)	*0.6*
RAI ablation				
Yes	0.35 (0.2–0.7)		0.30 (0.2–0.8)	
No	1.09 (1.0–1.9)	*<0.0001*	1.00 (0.6–1.8)	*<0.0001*

*I-131* radioactive iodine 131, *RR* relative risk, *CI* confidence interval, *ETE* extra-thyroidal extension, *LVSI* lymphovascular space invasion, *TG* thyroglobulin, *RAI* radioactive iodine

## Discussion

Despite excellent DFS rates in patients with PMC, about 3–16 % of patients develop local and distant failures [11]. In present study, we were able to determine overall five and ten year DFS rates of 94.7 % and 89.6 % respectively after aggressive treatment by total thyroidectomy followed by RAI ablation in the majority of cases. These results were found in consistent with similar reported data [12–15]. Several clinicopathological and treatments related prognostic factors were observed. An important prognostic factor, the age > 45 years was not found a prognosticator to predict DFS in our study, suggesting that other risk factors, such as aggressive histopathologic variants, multifocality, ETE, and LVSI are more important clinicopathological predictors than age in PMC [16]. Similarly, in contrast to other reported data, gender was also not found an important predictor of DFS [17]. Improved DFS was observed in patients who underwent total thyroidectomy. Possible explanation for this could be (a) high percentage of multifocality, and (b) more aggressive histopathological variants (tall cell and diffuse sclerosing variants) in our series, which is in agreement with few previously published studies of PMC [18–20].

Recent studies regarding PMC have reported that patients with multinodular goiter (MNG) and with lymphocytic or Hashimotos’ thyroiditis are associated with better prognosis; however, we could not reproduce the same results. Reason could be (a) few cases of histopathological proven MNG (32.5 %); (b) lack of preoperative TFTs in MNG patients; and (c) few number of patients with lymphocytic/Hashimoto’s thyroiditis (37.5 %) [21].

Further, present study showed the lymph node involvement and tumor size as the most significant independent risk factors for recurrence. Although we found tumor size > 0.5 cm seem to be associated with high recurrence rates, we were not able to identify a size threshold below which there was no lymph node involvement and no risk of recurrence; as in tumor of size ≤ 0.5 cm, 9.3 % lymph node metastasis were seen along with 2.5 % local and 1.3 % distant failures. This supports the hypothesis, that lymph node involvement status is higher in PMC of size > 0.8 cm, but is independent of tumor size [22]. Patients tolerated adjuvant RAI ablation very well with minimal toxicity. Failure of RAI ablation to decrease local or distant failure risk in N1a/b as compared to N0 disease is an indicator of underlying tumor burden in neck and this supports the idea of prophylactic central neck dissection during thyroidectomy [23]. However, still there is much debate over the prophylactic central neck dissection because of potential increased risk of hypoparathyroidism associated with central neck dissection [24].

Strengths of our study were; (a) reasonable sample size of Saudi patients with PMC, and (b) long term follow up period. Limitations of our study were; (a) retrospective data; (b) no intention to treat based analysis, and (c) lack of availability of preoperative clinical data, diagnostic methods (FNAC and radiology), tumor characteristics and baseline TFTs.

## Conclusions

In conclusion, among all PTC, 31.6 % of patients are diagnosed as PMC. Despite excellent DFS rates, a small proportion of patients with PMC develop recurrences after treatment. These recurrences not only badly affect physical health, but also mental and social health and overall quality of life. Based on our results we conclude that;

- High percentage of multifocality in our population of PMC favors near total or total thyroidectomy against lobectomy, which can be an option for unifocal PMC.
- Age > 45 years and gender were not found strong prognostic factors of DFS.
- Adjuvant RAI therapy improves DFS in PMC patients with aggressive histopathologic variants, multifocality, ETE, LVSI, tumor size (>0.5 cm in absence of other features) and lymph node involvement (≥150 mCi). In absence of N0 neck, there is significant difference of DFS in two doses (30 mCi vs. 100 mCi).
- Failure of RAI ablation to decrease risk in N1a/b supports prophylactic central neck dissection during thyroidectomy, however more trials are warranted.

## Abbreviations

DTC: Differentiated thyroid cancer
DFS: Disease free survival
DMC: Distant metastasis control
ETE: Extrathyroid extension
PMC: Papillary microcarcinoma
PTC: Papillary thyroid cancer
FTC: Follicular thyroid cancer
LVSI: Lymphovascular invasion
LR: Locoregional recurrence
LRC: Locoregional control
mCi: Millicurie
OS: Overall survival
RAI: Radioactive iodine-131
TG: Thyroglobulin
WBS: Whole body scintigraphy
