# Erratum to: Adjuvant Radioactive iodine 131 ablation in papillary microcarcinoma of thyroid: Saudi Arabian experience

**DOI:** 10.1186/s40463-016-0122-x

**Published:** 2016-01-29

**Authors:** Khalid Hussain AL-Qahtani, Mushabbab Al Asiri, Mutahir A. Tunio, Naji J. Aljohani, Yasser Bayoumi, Hanadi Fatani, Abdulrehman AlHadab

**Affiliations:** Department of Otolaryngology-Head & Neck Surgery, College of Medicine, King Saud University, Riyadh, Saudi Arabia; Radiation Oncology, Comprehensive Cancer Center, King Fahad Medical City, Riyadh, 59046 Saudi Arabia; Endocrinology and thyroid Oncology, King Fahad Medical City, Riyadh, 59046 Saudi Arabia; Radiation Oncology, NCI, Cairo University, Cairo, Egypt; Histopathology, King Fahad Medical City, Riyadh, 59046 Saudi Arabia; Radiation Oncology, King AbdulAziz University, Riyadh, 59046 Saudi Arabia

Unfortunately, the original version of this article [1] contained two errors. The title incorrectly referred to “radioactive iodine 133” instead of “radioactive iodine 131” and contained unnecessary quotation marks.

The title has been updated in the original article and is also correctly included in full in this erratum.
